# Posterior Reversible Encephalopathy Syndrome in a Patient of Sepsis-induced Cardiomyopathy, Successfully Managed with Intra-aortic Balloon Pump

**DOI:** 10.5005/jp-journals-10071-23152

**Published:** 2019-04

**Authors:** Anudeep Saxena, Vivek Bhargava, Aditya Shreya, Anshul Patodia, Pradeep Goyal

**Affiliations:** 1,2 Department of Anesthesiology, Narayana Multispeciality Hospital, Jaipur, Rajasthan, India.; 3 Department of Gastrointestinal Surgery, Narayana Multispeciality Hospital, Jaipur, Rajasthan, India.; 4 Department of Cardiology, Narayana Multispeciality Hospital, Jaipur, Rajasthan, India.; 5 Department of Cardiac Anesthesiology, Narayana Multispeciality Hospital, Jaipur, Rajasthan, India.

**Keywords:** Intra-aortic balloon pump, Posterior reversible encephalopathy syndrome, Septic cardiomyopathy

## Abstract

In patients with septic shock refractory to pharmacological agents, mechanical devices have been used successfully, although the reports are scarce. We report a case of septic shock where intra-aortic balloon pump (IABP) initiation leads to drastic improvement and survival from severe septic cardiomyopathy when conventional therapy was not effective. A 19-year-old male patient underwent surgery for adenocarcinoma descending colon. On day 8 he was reoperated for anastomotic leak and developed severe cardiomyopathy associated with septic shock, postoperatively. When he was in a vicious cycle of refractory hypotension, metabolic acidosis and severe cardiomyopathy, IABP was instituted along with other management for septic shock. Over next 3 days patient's hemodynamics improved and IABP was weaned off. While recovering from shock he developed posterior reversible encephalopathy syndrome which was promptly managed. This case report emphasizes on early institution of IABP in case of severe left ventricular dysfunction in septic shock.

**How to cite this article:** Saxena A, Bhargava V, *et al*. Posterior Reversible Encephalopathy Syndrome in a Patient of Sepsis-induced Cardiomyopathy, Successfully Managed with Intra-aortic Balloon Pump. Indian J Crit Care Med 2019;23(4):188–190.

## INTRODUCTION

Sepsis-induced cardiomyopathy is a complication of severe sepsis and septic shock described as a reversible myocardial depression that occurs in patients with septic shock. Sepsis-induced cardiomyopathy has three characteristics: left ventricular dilatation, depressed ejection fraction, and recovery in 7–10 days.^1^ Incidence of sepsis-induced left ventricular hypokinesia has been reported to be as high as 60%.^2^ Use of IABP has been reported to be lifesaving in certain sepsis-induced cardiomyopathy.^3,4^ We report a case with IABP introduction leading to drastic improvement, and survival from severe septic cardiomyopathy when conventional therapy was not effective, also how the course in the hospital was complicated by posterior reversible encephalopathy syndrome (PRES).

## CASE REPORT

**Fig. 1:**
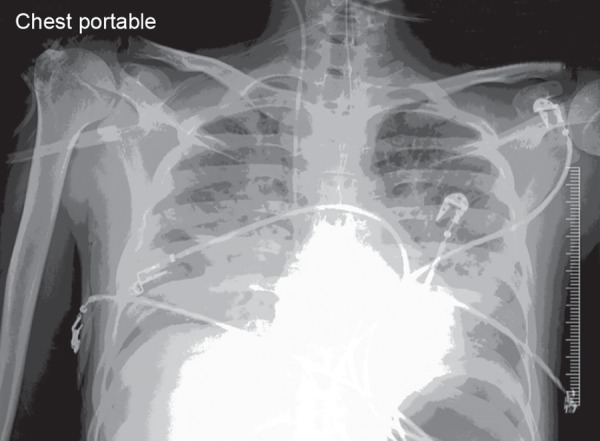
X-ray chest of patient on day 2 of intensive care unit (ICU) admission when he developed pulmonary edema.

A 19-year-old Indian male was admitted with complains of abdominal pain, nausea, vomiting, and weight loss. He was diagnosed with moderately differentiated adenocarcinoma (T2N0M0) and underwent left hemicolectomy, colocolic, and jejunojejunal anastomosis. Seven days postoperatively patient developed anastomotic leak for which he was reoperated. Postoperatively (day 2 of ICU), patient required high dose vasopressor, his condition worsened and had to be put on mechanical ventilation for pulmonary edema (Fig. 1). 2D-echocardiography revealed severely depressed left ventricular function.

Echo findings are as follows: Global hypokinesia more in anteroseptal wall, anterior wall, and intraventricular septum. Left ventricular ejection fraction (LVEF) is 15–20% (visually estimated). Mild mitral regurgitation, mild tricuspid regurgitation, no pulmonary artery hypertension, intra-ventricular and intra-atrial septum are intact. No pericardial effusion, clot or vegetation seen.

ECG showed sinus tachycardia (heart rate—150 bpm), poor R wave progression and nonspecific ST-T changes. Troponin I 1.69 ng/mL, CPK-MB 5.2 ng/mL, BNP 2000 pg/mL.

Possibility of acute coronary syndrome was kept as a differential, but possibility of septic myocarditis was strongly considered. Patient was started on daily aspirin 75 mg, clopidogrel 75 mg, injection fondaparinux 2.5 mg, and injection torsemide 20 mg thrice daily.

Over the next 24 hours (day 3) patient's hypotension and metabolic acidosis worsened despite high dosage of epinephrine, norepinephrine and dobutamine as well as stress dose of hydrocortisone. His tachycardia was bothersome. He had chilled peripheries and seemed to be maximally vasoconstricted. His urine output started falling. Antibiotics were escalated according to pus culture sensitivity reports. Considering cardiogenic shock to be primarily responsible for the patient's condition, it was planned to put IABP. Without wasting time 34 Fr IABP catheter was inserted through right femoral artery. IABP support was initiated with the use of 1:1 electrocardiographic triggering. In few hours CVP was reduced from 18 mm Hg to 10 mm Hg and heart rate settled from 160 bpm to 120 bpm.

**Fig. 2:**
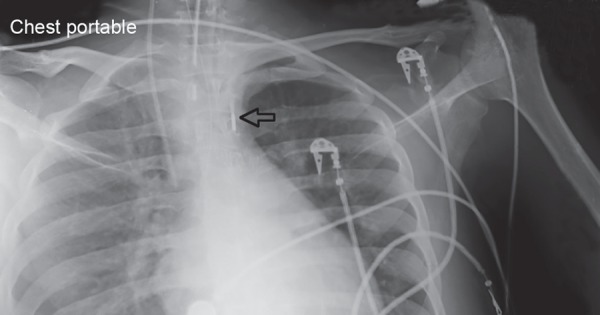
X-ray chest of patient on day 6. Intra-aortic balloon pump can be seen just distal to arch of aorta. Clearing of pulmonary edema as compared to the previous X-ray can be noted.

**Fig. 3:**
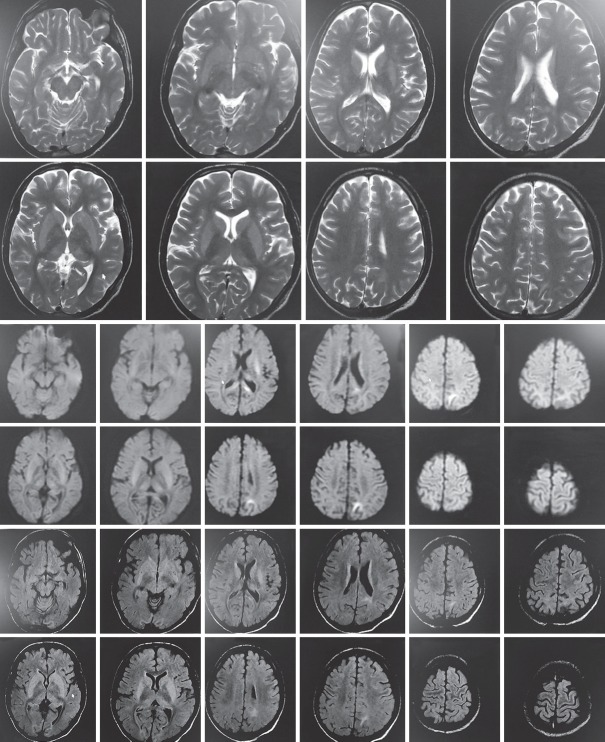
There is presence of fairly bilaterally symmetrical, ill defined, mild T2, and FLAIR hyperintensity in bilateral basal ganglia and thalamus. In the splenium of corpus callosum and in the cortical–subcortical aspect of bilateral occipital lobes and left posterior parietal lobe there is mild DWI hyperintensity with corresponding mild hypointensity on ADC images (predominantly in the splenium of corpus callosum and in subcortical aspect of left posterior parietal lobe), suggesting diffusion restriction.

Over next 48 hours patient's hemodynamic improved. Lungs were clearer (Fig. 2). Heart rate started settling from 120 bpm to around 100 bpm. Ionotropic supports were gradually tapered maintaining a mean arterial pressure of 65 mm Hg. Patient started pouring good urine output. On day 6 (day 3 of starting IABP), platelet counts started falling. Patient was maintaining his blood pressure at minimum dose of epinephrine and norepinephrine. IABP was removed. Till next day, when the effect of atracurium was completely weaned off, patient did not become alert. He was opening eyes spontaneously but was not following command; gaze was upward, bilateral extensor planter reflex. MRI brain was done which showed symmetrical mild ill-defined hyperintensity in bilateral occipital lobes, basal ganglia-thalamic region and splenium of corpus callosum (Fig. 3). This was suggestive of possibility of PRES.

On day 9 of ICU admission patient's tracheostomy was done. Patient continued to be on vasopressor and inotropes, although requirement was low. Antibiotics were further modified according to blood and other fluid culture reports. Over next 5 days epinephrine and norepinephrine were completely withdrawn. Patient neurologically improved and started following commands. After pressure support trial for a day, on day 16 of ICU admission his ventilator support was withdrawn. On day 18, patient's repeat 2D echo was performed which showed an improved LVEF of 40–45% with mild septal hypokinesia. He was shifted out of ICU.

His trachea was decannulated and on day 21 he was discharged from the hospital with functioning colostomy in situ.

## DISCUSSION AND CONCLUSION

In sepsis, both the peripheral oxygen requirement and cardiac output are generally increased. However, in patients with septic cardiomyopathy, the cardiac output becomes inadequate to supply the increased systemic and myocardial oxygen requirement. Metabolic acidosis further impairs myocardial contractility; patient enters in a vicious circle and eventually succumbs. The two primary benefits of IABP are augmented coronary perfusion and reduced LV afterload thereby increased cardiac output.

Our idea of initiating IABP was to buy time for the antibiotics to start acting. So that the oxygen requirement of the patient comes down to normal, that could be sustained by a compromised heart.

The time of initiating IABP is particularly important as shown previously by Ogunbayo G et al.^5^ They have concluded that early use of IABP (within 24 hours of presentation) appears to offer a mortality benefit. In our patient we initiated IABP within 12 hours of identification of cardiomyopathy and LV dysfunction.

Posterior reversible encephalopathy syndrome is commonly, but not always associated with acute hypertension.^6^ In a retrospective study, Bartynski et al. reported an association of PRES with infection, sepsis and shock in 26.3% (25 out of 106) patients.^7^ In our patient there was no episode of hypertension. In sepsis, an alteration in vascular tone develops secondary to competing vasoconstrictive (platelet degranulation with thromboxane release, endothelin-1, angiotensin, vasopressin, and central sympathetic stimulation) and vasodilatory (nitric oxide, prostacyclin) effects.^8,9^

We suggest that in a patient of septic shock with cardiomyopathy, if pharmacologic agents are insufficient in maintaining systemic perfusion, then IABP should be considered promptly, particularly in patients with severe LV dysfunction (LVF <30%). Further elaborated trials are required before these mechanical devices would probably establish their place in future sepsis guidelines.
